# The expression and functional role of proline-rich 15 in non-small cell lung cancer

**DOI:** 10.1038/s41419-025-07373-x

**Published:** 2025-02-10

**Authors:** Yong Ji, Han Zhang, Fei-long Gong, Jia-long Liang, Sheng-fei Wang, Yong-hua Sang, Ming-feng Zheng

**Affiliations:** 1 Department of Thoracic Surgery, The Affiliated Wuxi People’s Hospital of Nanjing Medical University, Wuxi People’s Hospital, Wuxi Medical Center, Nanjing Medical University, Wuxi, China; 2 Department of Medical Oncology, Harbin Medical University Cancer Hospital, Harbin Medical University, Harbin, Heilongjiang China; 3 Department of Cardiothoracic Surgery, The Second Affiliated Hospital of Soochow University, Suzhou, China

**Keywords:** Oncogenes, Non-small-cell lung cancer

## Abstract

Proline-rich 15 (PRR15) is a protein primarily known for its role in placental development. This study investigates the expression, functional significance, and underlying mechanisms of PRR15 in non-small cell lung cancer (NSCLC). Our findings demonstrate significantly elevated *PRR15* expression in NSCLC tissues compared to normal lung parenchyma, with higher expression correlating with adverse clinical outcomes. Single-cell RNA sequencing confirmed *PRR15* overexpression within the malignant tumor cell population. PRR15 expression was elevated in NSCLC tissues from locally treated patients and in a panel of primary and established NSCLC cells. PRR15 depletion using shRNA or CRISPR/Cas9-mediated knockout significantly suppressed proliferation and migration, while promoting apoptosis in various NSCLC cells. Conversely, ectopic PRR15 overexpression using a lentiviral construct enhanced cell proliferation and migration. Mechanistic investigations implicated PRR15 in the activation of the Akt-mTOR signaling pathway. Inhibition of PRR15 expression via shRNA or CRISPR/Cas9-mediated knockout resulted in decreased Akt and S6K phosphorylation, while PRR15 overexpression led to augmented Akt-S6K signaling in primary human NSCLC cells. In vivo studies using xenograft models further validated the oncogenic role of PRR15, demonstrating that PRR15 knockdown suppressed tumor growth and attenuated Akt-mTOR activation. These findings collectively highlight the potential of PRR15 as a novel oncogenic driver and therapeutic target in NSCLC.

## Introduction

Non-small cell lung cancer (NSCLC) accounts for about 85% of all lung cancer cases and includes histological types of adenocarcinoma (LUAD), squamous cell carcinoma (LUSC), and large cell carcinoma (LCLC) [1]. NSCLC arises from the lung epithelial cells and is driven by a mix of genetic factors and environmental influences, such as exposure to tobacco smoke and other carcinogens [2, 3]. Treatments for NSCLC involve various strategies, including surgery, radiation, chemotherapy, targeted therapies, and immunotherapy [4, 5]. Advances in molecular profiling have uncovered key mutations and signaling pathways, facilitating the development of targeted treatments [6–8]. The late diagnosis of NSCLC, often leading to a poor prognosis [9, 10], underscores the urgent need for improved methods for early detection and innovative treatments [11, 12].

Molecularly-targeted agents have transformed the management of NSCLC by targeting specific genetic alterations [6, 8, 13, 14]. Epidermal growth factor receptor (EGFR) inhibitors, including erlotinib, gefitinib, and osimertinib, target prevalent EGFR mutations [15, 16]. Anaplastic lymphoma kinase (ALK) inhibitors, such as crizotinib, alectinib, and lorlatinib [17, 18], as well as ROS1 (c-ros oncogene 1) inhibitors (i.e., crizotinib and entrectinib [19]) are effective against certain NSCLC patients. BRAF inhibitors, particularly the combination of dabrafenib and trametinib, can target BRAF V600E mutations in NSCLC [6]. MET inhibitors including capmatinib and tepotinib target MET exon 14 skipping mutations, while RET inhibitors such as selpercatinib and pralsetinib focus on RET gene fusions in NSCLC [20, 21]. Similarly, sotorasib, a KRAS inhibitor, offers a targeted option for the KRAS G12C mutation in NSCLC [22, 23]. These tailored treatment approaches, based on specific signaling targets, hold promise for improved outcomes in NSCLC [6, 8].

During early pregnancy, the nuclear protein proline-rich 15 (PRR15), produced by trophoblast cells, plays a critical role in ensuring proper embryonic development and formation of the placenta [24]. PRR15 is predicted to contain four potential phosphorylation sites for protein kinase C (PKC), two sites for tyrosine kinase II phosphorylation, as well as a sequence targeting the nucleus [25]. PRR15 silencing in trophoblasts resulted in embryo loss, hindering trophoblast gene expression, proliferation, survival, focal adhesion, and also affected p53 signaling [24]. PRR15 expression is regulated by glycogen synthase kinase 3 beta (GSK3β) and GSK3β silencing inhibited transcription and expression of PRR15 in trophoblasts [26].

It is important to note that there are limited studies examining the role of PRR15 in cancer. Research on PRR15 expression in gastrointestinal (GI) cancers revealed varying levels in mouse GI tumors and human colorectal cancer (CRC) as a result of diverse gene mutations [27]. In mouse GI tumors, increased PRR15 expression was linked to mutations in the AP (adenomatous polyposis coli) gene, similar to findings in human CRC [27]. PRR15 expression was elevated in breast cancer, accompanied by reduced gene methylation in tumors [28]. PRR15 exhibits a complex role in breast cancer, inhibiting epithelial-to-mesenchymal transition while simultaneously activating estrogen receptor signaling, both of which contribute to tumor progression [28]. Furthermore, elevated PRR15 expression has been observed in colon adenocarcinoma compared to normal tissue [29]. To date, the specific function of PRR15 in NSCLC has not been elucidated. This study aims to fill this critical gap by investigating PRR15 expression levels and exploring its potential functional implications in NSCLC. Through this research, we aim to enhance the understanding of the molecular mechanisms involved in NSCLC and identify potential new therapeutic targets.

## Materials and methods

### Reagents and antibodies

Puromycin, cell culture medium, fetal bovine serum (FBS), Matrigel matrix, polybrene and cell counting kit-8 (CCK-8) were procured from Sigma-Aldrich (St. Louis, MO). Fluorescent labeling reagents, including 5-ethynyl-2’-deoxyuridine (EdU), terminal deoxynucleotidyl transferase dUTP nick end labeling (TUNEL), DAPI (4’,6-diamidino-2-phenylindole) and JC-1 were acquired from Thermo-Fisher Invitrogen (Suzhou, China). All primary antibodies utilized in these studies were sourced from Cell Signaling Technology (Danvers, MA, USA) and Thermo-Fisher Invitrogen (Suzhou, China). Unless otherwise indicated, viral vectors, nucleotide sequences, and PCR primers were synthesized by Genechem (Shanghai, China).

### Cells

A549 NSCLC cell line, provided by Dr. Xu at Soochow University [30–33], was cultured in high-glucose DMEM supplemented with 10% fetal bovine serum (FBS). Additionally, primary NSCLC cells (pNSCLC-1, pNSCLC-2, and pNSCLC-3) derived from three written-informed consent patients, along with primary human lung epithelial cells (pEpi1 and pEpi2) from two written-informed consent patients, were also sourced from Dr. Xu [30–33]. The primary NSCLC cells exhibited *PTEN* depletion and mutations in *PI3KCA* and *KRAS*. All procedures adhered to the Declaration of Helsinki principles and received approval from the Ethics Committee of The Affiliated Wuxi People’s Hospital of Nanjing Medical University (WYYBS-2022-078). Monthly checks for mycoplasma and microbial contamination were conducted, with routine verification of cell doubling time, colony-forming efficiency, and morphology.

### Human tissues

Provided by Dr. Xu at Soochow University [30, 33], a total of 23 patients diagnosed with primary NSCLC participated in this study. Patient demographics were as follows: 10 females and 13 males, with ages ranging from 42 to 71 years, and tumor stages III to VI. All patients provided written-informed consent for tissue collection. Freshly resected cancerous tissues and adjacent normal lung tissues were utilized for immediate analysis [30, 33]. The study protocols adhered to the ethical principles outlined in the Declaration of Helsinki and were approved by the Ethics Committee of The Affiliated Wuxi People’s Hospital of Nanjing Medical University.

### Western Blotting

Tissues and cells were lysed using radioimmunoprecipitation assay buffer (Beyotime, Wuxi, China) supplemented with protease inhibitors (Beyotime, Wuxi, China). Protein concentration was determined with a BCA (bicinchoninic acid) kit (Thermo-Fisher Invitrogen, Shanghai, China). Proteins were separated on 10–12.5% sodium dodecyl sulfate–polyacrylamide gels and transferred to PVDF membranes (Millipore, Burlington, MA). The membranes were blocked and then incubated with primary antibodies overnight, followed by a 50-min incubation with secondary antibodies. Detection of target protein bands was performed using the ECL system. Fig. S1 listed the un-cropped blotting images.

### Quantitative real-time polymerase chain reaction (qPCR)

Total RNA of cultured cells or tissues was isolated via TRIzol reagent (Takara, Otsu, Japan). RNA concentration was measured by UV Spectrophotometry at the absorbance of 260 nm (A260), and cDNA was synthesized with a Reverse Transcription Kit (Applied Biosystems, Foster City, CA). The qPCR reactions were conducted using the SYBR Green real-time PCR Kit (Takara, Tokyo, Japan) on a Bio-Rad system (Bio-Rad, Hercules, CA). mRNA levels were assessed using the 2^−ΔΔCt^ method, with *GAPDH* (*glyceraldehyde-3-phosphate dehydrogenase*) serving as the reference gene.

### PRR15 shRNA

To achieve PRR15 knockdown, shRNA experiments were conducted on NSCLC cells and lung epithelial cells cultured in complete medium with polybrene at 50% confluence. Lentiviral particles containing PRR15 shRNA sequence, in GV369 construct, obtained from Genechem (Shanghai, China), were introduced into the cells at a multiplicity of infection (MOI) of 10. Following a 42 h infection period, cells were exposed to a medium containing puromycin (2–3 μg/mL) to select for stable colonies over six subsequent passages. Two specific shRNAs were utilized, including shPRR15-1# and shPRR15-2#, targeting non-overlapping sequences. Control cells were infected with lentiviral particles containing scramble shRNA (shC, Genechem). *PRR15* mRNA and protein levels in stable cells were monitored regularly.

### CRISPR/Cas9-induced PRR15 knockout (KO)

Gene editing for PRR15 was conducted in primary human NSCLC cells (pNSCLC-1) grown in polybrene-containing medium at 60% cell density. Cells were first infected with the Cas9-expressing lentivirus (provided by Dr. Cao [34]) and stable cell clones were selected. Subsequently, these cells were transduced with lentivirus containing a CRISPR/Cas9-PRR15-KO construct with PRR15-targeting sgRNA from Genechem (Shanghai, China). Following puromycin selection, stable cell clones were isolated and seeded into 96-well plates at single-cell densities. Sequencing of the PRR15 locus verified gene disruption and KO status was further confirmed through Western Blotting assays. Two stable PRR15 KO single-cell-derived colonies (“koPRR15-Cln1” and “koPRR15-Cln2”) were created. Control cells were treated with lentivirus carrying a CRISPR/Cas9-empty control vector (“koC”), also provided by Genechem.

### PRR15/HER2 (human epidermal growth factor receptor 2) overexpression

To achieve PRR15/**HER2** overexpression, NSCLC cells were treated with lentivirus containing a PRR15-expressing vector (in GV369 construct, provided by Genechem) at MOI of 12. This vector included the PRR15 cDNA sequence or HER2 cDNA sequencewithout any tags, and the infection period was 36 h. Afterward, cells were maintained in puromycin-containing complete medium to create stable cells after f 5–6 passages. The mRNA and protein levels of PRR15 or HER2 in these stable cells were routinely assessed. Control cells were transduced with the empty vector.

### CCK-8 assay

Cell viability was assessed using the CCK-8 assay. Specifically, 3000 cells per well were seeded in a 96-well plate and cultured for 72 h. Subsequently, 25 μL of CCK-8 reagent was added to each well and incubated for 2.5 h. Cell viability, reflected in the reduction of the CCK-8 reagent, was quantified by measuring absorbance at 450 nm using a microplate reader.

### Colony formation assay

For colony formation assays, cells (at 12,000 cells per dish), with applied genetic treatments, were seeded in 10-cm diameter culture dishes. After incubation for 10 days, colonies were fixed, stained, and counted manually.

### The nuclear EdU/TUNEL staining assays

Briefly, NSCLC cells or lung epithelial cells, with designated genetic modifications, were grown in 12-well plates for specified durations. They were then fixed with paraformaldehyde and permeabilized with Triton X-100. TUNEL/EdU and DAPI dyes were applied for staining. Images of the stained cells were captured using a Zeiss fluorescence microscope. The proportion of EdU/TUNEL-positive cells to DAPI-stained nuclei was determined by analyzing five randomly chosen fields.

### “Transwell” assays

Briefly, the specific NSCLC cells, at 8000 cells per insert, were placed on the top side of “Transwell” inserts and allowed to migrate for 24 h. Cells that reached the bottom surface were fixed and stained with crystal violet.

### Caspase-3/9 activity assay

Caspase-3 and Caspase-9 activities were determined using colorimetric assay kits (Thermo-Fisher Invitrogen, Suzhou, China) based on manufacturer’s protocols. Briefly, cell/tissue lysates (1 μg/μL, 20 μg per sample) were prepared and incubated with specific caspase substrates. Enzymatic cleavage of these substrates generated a colorimetric product, which was quantified spectrophotometrically at 405 nm. A standard curve was always constructed to calculate caspase activity.

### JC-1 staining

Mitochondrial membrane potential (MMP) was assessed using the JC-1 assay kit (Sigma) according to the manufacturer’s protocols. Cells were incubated with JC-1 dye under specified conditions. This cationic dye exhibits potential-dependent accumulation within mitochondria. Cells with the applied genetic treatments were incubated with JC-1 dye (5.0 μg/mL) for 30 min, and excess dye was removed by washing. The resulting green fluorescence intensity, indicative of decreased MMP, was quantified at an excitation wavelength of 490 nm using fluorescence microscopy.

### Detection of cytosol cytochrome c contents

Cytochrome c levels were quantified using an enzyme-linked immunosorbent assay (ELISA) from Thermo-Fisher Invitrogen (Suzhou, China). The assay involves immobilizing capture antibodies specific to cytochrome c onto a microplate. The cytosol protein lysates (1 μg/μL, 20 μg per sample) containing cytochrome c were added to the plate, binding to the capture antibody attached in the kit. Subsequently, a detection antibody conjugated to an enzyme is introduced, forming a complex with captured cytochrome c. Addition of a substrate solution resulted in an enzyme-mediated colorimetric reaction, which was detected at 450 nm. A standard curve was generated to determine cytochrome c concentrations based on the obtained absorbance values.

### Constitutively active mutant Akt1 (caAkt1)

Primary human NSCLC cells were transduced with the lentiviral vector encoding the constitutively-active S473D mutant of Akt1 (caAkt1, from Dr. Cao [35, 36]). Following puromycin selection, stable caAkt1-expressing NSCLC cells were established. Control cells were treated with the corresponding empty vector (“Vec”), also from Dr. Cao.

### Xenograft assays

Nude BALB/c mice, each weighing between 17.9 and 18.4 g and of both genders, were sourced from Shanghai SLAC Laboratory Animal Co. (Shanghai, China). Eight million genetically-modified primary NSCLC cells were subcutaneously (*s.c*.) injected into the mice’s flanks. Monitoring of subcutaneous tumor growth began on Day-14. Tumor volumes, body weights, and daily tumor growth rates (measured in mm³ per day) were recorded according to established guidelines [30, 31]. Immunohistochemistry (IHC) was performed on paraffin-embedded tissue Section (4 μm sickness). Following standard deparaffinization and rehydration procedures, antigen retrieval was conducted using citrate buffer. To block endogenous peroxidase activity, tissue sections were incubated with hydrogen peroxide. Subsequently, a Ki-67 staining kit (Biyuntian, Wuxi, China) was utilized. Additionally, TUNEL staining (using a Biyuntian kit, Wuxi, China) was performed in the tumor slides, followed by washing and counterstaining with DAPI to highlight cell nuclei. The sections were mounted with anti-fade medium and analyzed using a fluorescence microscope. All animal procedures received approval from the Institutional Animal Care and Use Committee (IACUC) and the Ethics Review Board of The Affiliated Wuxi People’s Hospital of Nanjing Medical University.

### Statistical analyses

All in vitro experiments were performed in five replicates. Data conforming to a normal distribution are presented as mean ± standard deviation (SD). Statistical analyses were conducted using SPSS software (Version 23.0 IBM Corp., Armonk, NY). Unpaired Student’s *t*-tests were employed for comparisons between two groups, while one-way analysis of variance (ANOVA) with post-hoc Scheffé's and Tukey’s multiple comparison tests were utilized for comparisons among three or more groups. A threshold of ***P*** < 0.05 was established for statistical significance.

## Results

### *PRR15* expression in NSCLC and clinical correlations

**Fig. 1:**
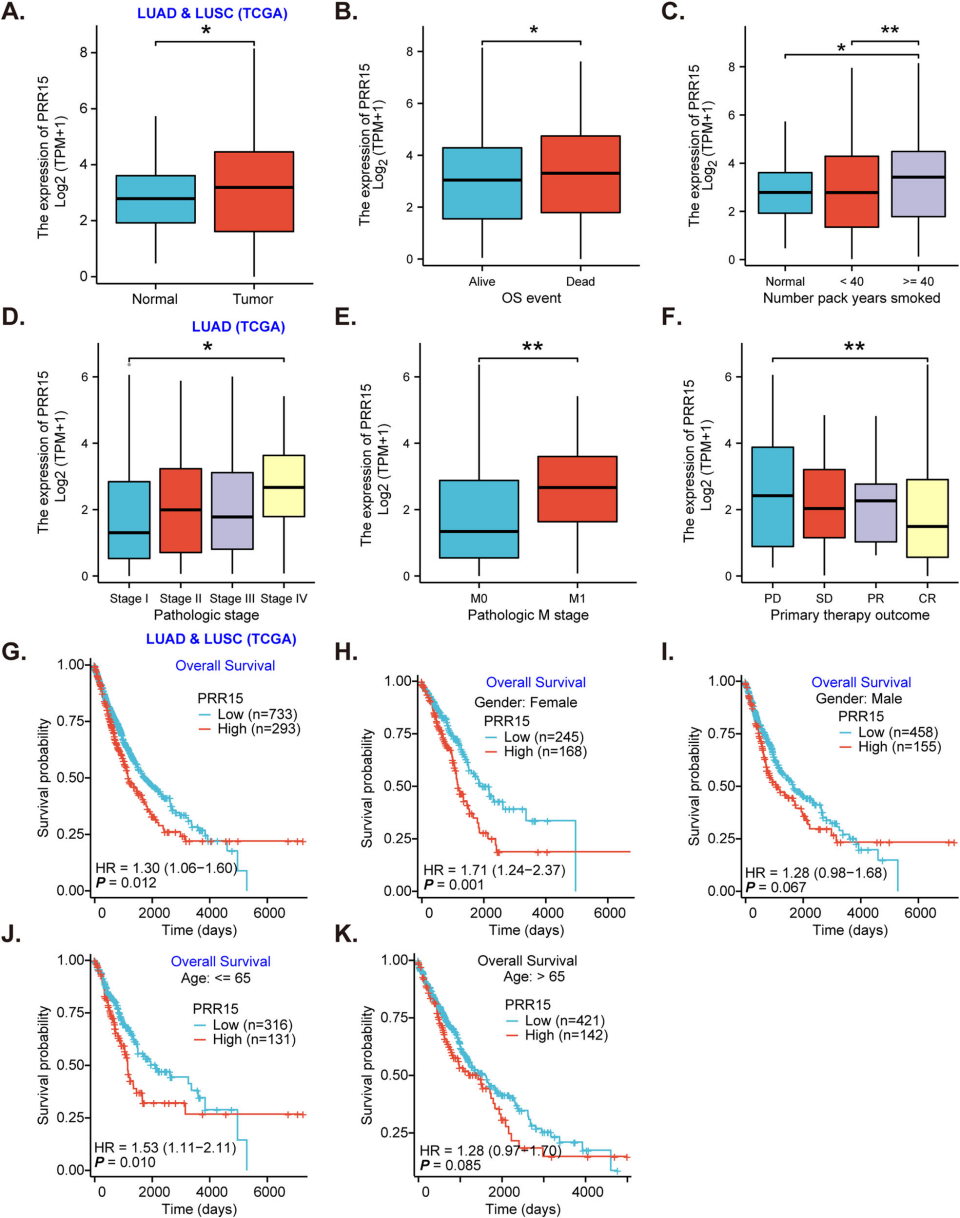
*PRR15* expression in NSCLC and clinical correlations. TCGA database shows *PRR15* expression in normal lung tissue and NSCLC (LUAD + LUSC) tissues (**A**). *PRR15* expression in NSCLC patients by survival status (**B**), smoking history (**C**), pathological tumor stage (**D**), metastatic status (**E**) or by treatment response (**F**). Kaplan–Meier survival curves for NSCLC patients stratified by *PRR15* expression and the described clinical factors (**G**–**K**). LUAD lung adenocarcinoma, LUSC lung squamous cell carcinoma, OS overall survival, PD progressive disease, SD stable disease, CR complete response, PR partial response. * indicates ***P*** < 0.05. ** indicates ***P*** < 0.01.

Analysis of TCGA (The Cancer Genome Atlas) data in NSCLC revealed significantly elevated *PRR15* levels in NSCLC [lung adenocarcinoma (LUAD) + squamous cell carcinoma (LUSC)] tumor tissues compared to normal lung parenchyma (Fig. 1A), suggesting a potential role for PRR15 in NSCLC pathogenesis. *PRR15* expression is significantly higher in patients who died from NSCLC than in patients who are alive (Fig. 1B). A positive correlation between *PRR15* expression and smoking history, with patients with a higher number of pack-years exhibiting significantly increased *PRR15* levels (Fig. 1C). Moreover, *PRR15* expression in NSCLC tissues is elevated in advanced disease stages (Fig. 1D) and metastatic NSCLC (Fig. 1E). Figure 1F indicates higher *PRR15* expression in patients with progressive disease compared to those with stable disease or response to therapy.

The prognostic significance of *PRR15* is also illustrated. Kaplan-Meier survival analysis demonstrated a significant association between high *PRR15* expression and decreased overall survival in the NSCLC cohort (LUAD + LUSC) (Fig. 1G). Moreover, in both female (Fig. 1H, *P* < 0.01) and male (Fig. 1I, *P* = 0.067) NSCLC patient subgroups, *PRR15* overexpression correlates with poor overall survival. Similarly, patients aged ≤65 years with high *PRR15* expression exhibited poorer survival compared to those with low *PRR15* expression (Fig. 1J, *P* = 0.01), while the trend was less pronounced in the >65 years group (Fig. 1K, *P* = 0.085). These findings collectively suggest that *PRR15* overexpression is associated with poor prognosis in NSCLC and may serve as a potential prognostic biomarker.

### Single-cell RNA sequencing analysis revealed PRR15 overexpression within the malignant cell population of NSCLC tumors

**Fig. 2:**
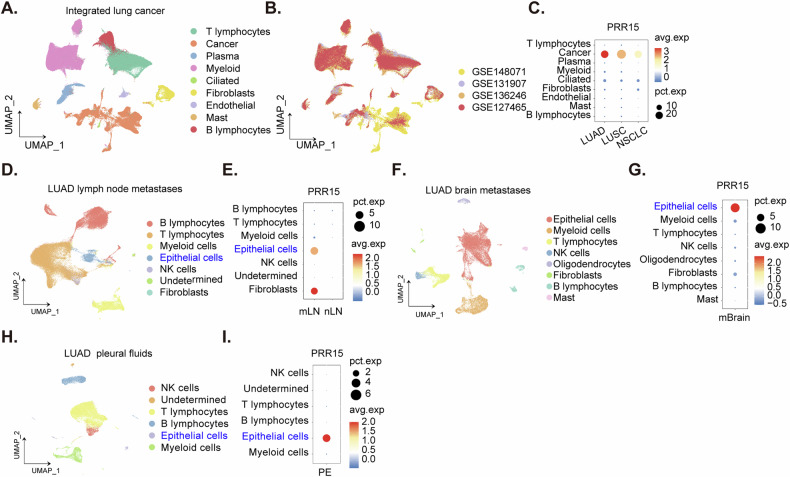
Single-cell RNA sequencing analysis revealed PRR15 overexpression within the malignant cell population of NSCLC tumors. Uniform Manifold Approximation and Projection (UMAP) visualization of cell annotations derived from single-cell RNA sequencing data of NSCLC demonstrates *PRR15* expression patterns (**A**, **B**). The distribution of *PRR15* is further quantified in a density plot (**C**). Analogous UMAP and density plot analyses of cell annotations from brain metastatic LUAD tissues (**D**, **E**), lymph node metastatic LUAD tissues (**F**, **G**), and LUAD pleural fluid (**H**, **I**) reveal the expression distribution of *PRR15*.

To investigate *PRR15* expression within the complex cellular landscape of NSCLC, we leveraged a publicly accessible single-cell RNA sequencing dataset. To ensure accurate cell type identification, we relied on cell annotations provided by the original study [37]. Dimensionality reduction analysis of these annotated cells revealed distinct clusters representing malignant cancer cells and various non-malignant cell types within the NSCLC tissue microenvironment (Fig. 2A). Additionally, we visualized the origin of integrated single-cell data using dimensionality reduction (Fig. 2B). Subsequent analysis of *PRR15* expression demonstrated significant *PRR15* overexpression in cancer cell populations compared to non-malignant cells, with particularly elevated levels observed in both LUAD and LUSC subtypes (Fig. 2C). These findings collectively indicate that *PRR15* is preferentially expressed in cancer cells within the NSCLC tumor microenvironment, suggesting its potential utility as a therapeutic target.

Single-cell sequencing analysis of lymph node metastatic (mLN) lung adenocarcinoma (LUAD) tissues (Fig. 2D) revealed overexpression of *PRR15* specifically within epithelial cell populations (Fig. 2D, E). Moreover, single-cell sequencing data from brain metastatic (mBrain) NSCLC (Fig. 2F) also demonstrated elevated *PRR15* expression in epithelial cell subsets of metastatic LUAD tissues (Fig. 2F, G). Furthermore, analysis of LUAD pleural effusion (PE) samples (Fig. 2H) confirmed the pronounced expression of *PRR15* within epithelial cell populations (Fig. 2H, I). Thus, multiple lines of evidence derived from single-cell sequencing and pleural fluid analyses consistently demonstrate overexpression of *PRR15* within cancer cell populations of metastatic LUAD.

### Increased PRR15 expression in NSCLC tissues of locally treated patients and various primary/established NSCLC cells

**Fig. 3:**
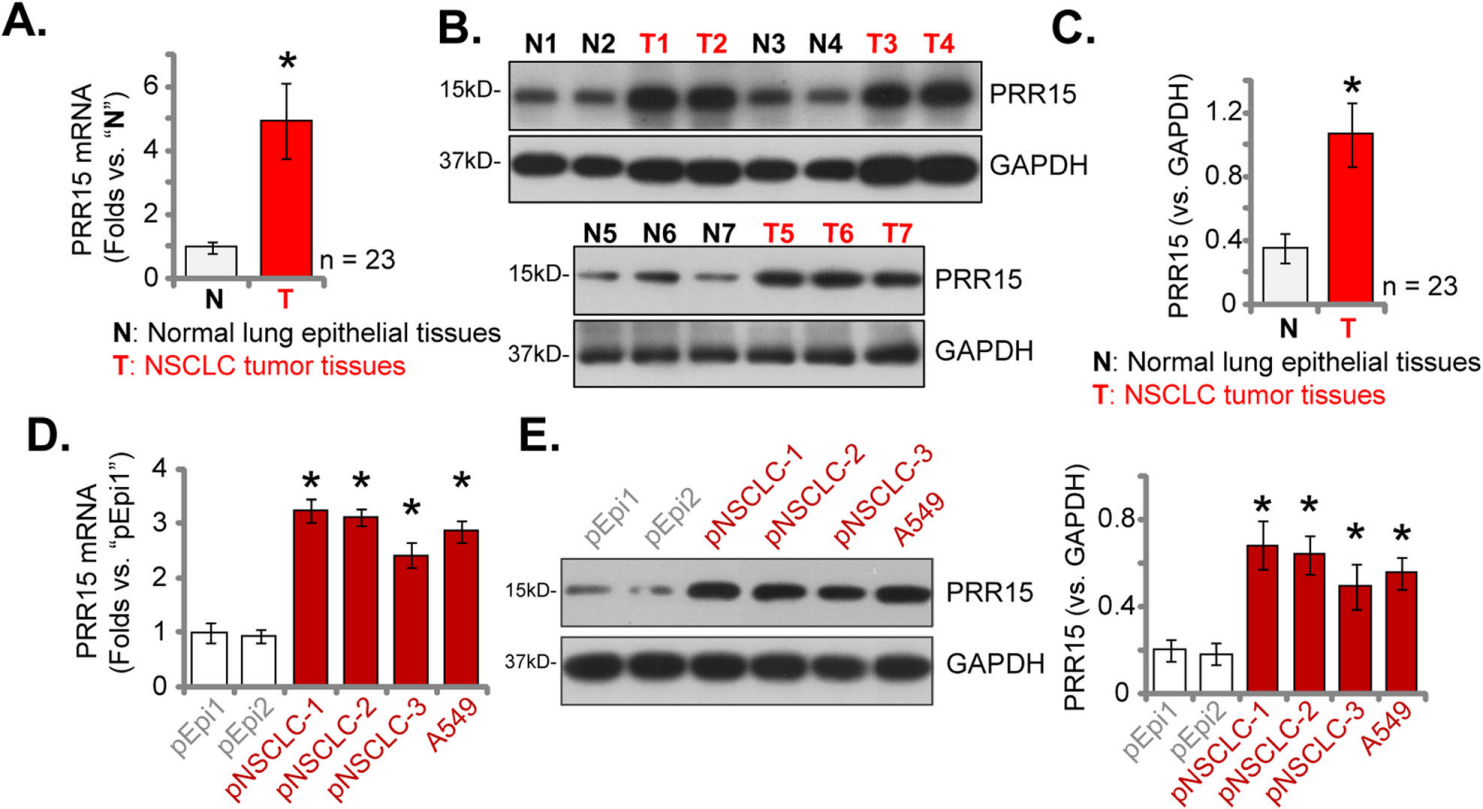
Increased PRR15 expression in NSCLC tissues of locally treated patients and various primary/established NSCLC cells. The levels of *PRR15* mRNA and protein were examined in NSCLC tumor samples (labeled as “T”) and the adjacent normal lung epithelial tissues (labeled as “N”) from 23 primary NSCLC patients (**A**–**C**). Furthermore, *PRR15* mRNA and protein levels were measured in the specified NSCLC cells and primary lung epithelial cells (**D**, **E**). The data values are presented as the mean ± standard deviation (SD). Statistical significance was marked by * ***P*** < 0.05 compared to the “N” tissues or “pEpi1” cells.

To further examine the PRR15 expression profile in locally treated NSCLC patient tissues, we carried out a detailed analysis. The NSCLC tumor samples (marked as “T”) and adjacent non-cancerous lung epithelial tissues (marked as “N”) from a group of 23 primary NSCLC patients, provided by Dr. Xu [30, 33], were collected. By analyzing the tissue lysates, we observed a significant increase in *PRR15* mRNA expression levels in the NSCLC tissues compared to the adjacent normal tissues (Fig. 3A). Additionally, the assessment of PRR15 protein expression showed an increase in tumor tissues from four representative patients (referred to as “T1” to “T7”) (Fig. 3B). While its expression in pared normal tissues was relatively low (referred to as “N1” to “N7”, Fig. 3B). Summarizing the data from all 23 patient samples, our findings indicated a statistically significant upregulation in PRR15 protein expression in the NSCLC tissues (Fig. 3C).

Follow-up experimental assessments were conducted to investigate PRR15 expression levels in different NSCLC cells. This included three different primary human NSCLC cells, specifically “pNSCLC-1/-2/-3”, also sourced from Dr. Xu [30–33], as well as the immortalized A549 cell line. The findings showed a significant increase in *PRR15* mRNA expression in both primary and immortalized NSCLC cells, compared to human lung epithelial cells, referred to as “pEpi1” and “pEpi2” from two separate donors [30–33] (Fig. 3D). Additionally, an upregulation of PRR15 protein was observed in all these primary and immortalized NSCLC cells (Fig. 3E), which was in stark contrast to the much lower expression observed in lung epithelial cells (Fig. 3E). These results further support the elevated expression of PRR15 in NSCLC tissues and cells, indicating a possible role for PRR15 in the pathogenesis of NSCLC.

### PRR15 shRNA triggers anti-cancer effects in NSCLC cells

**Fig. 4:**
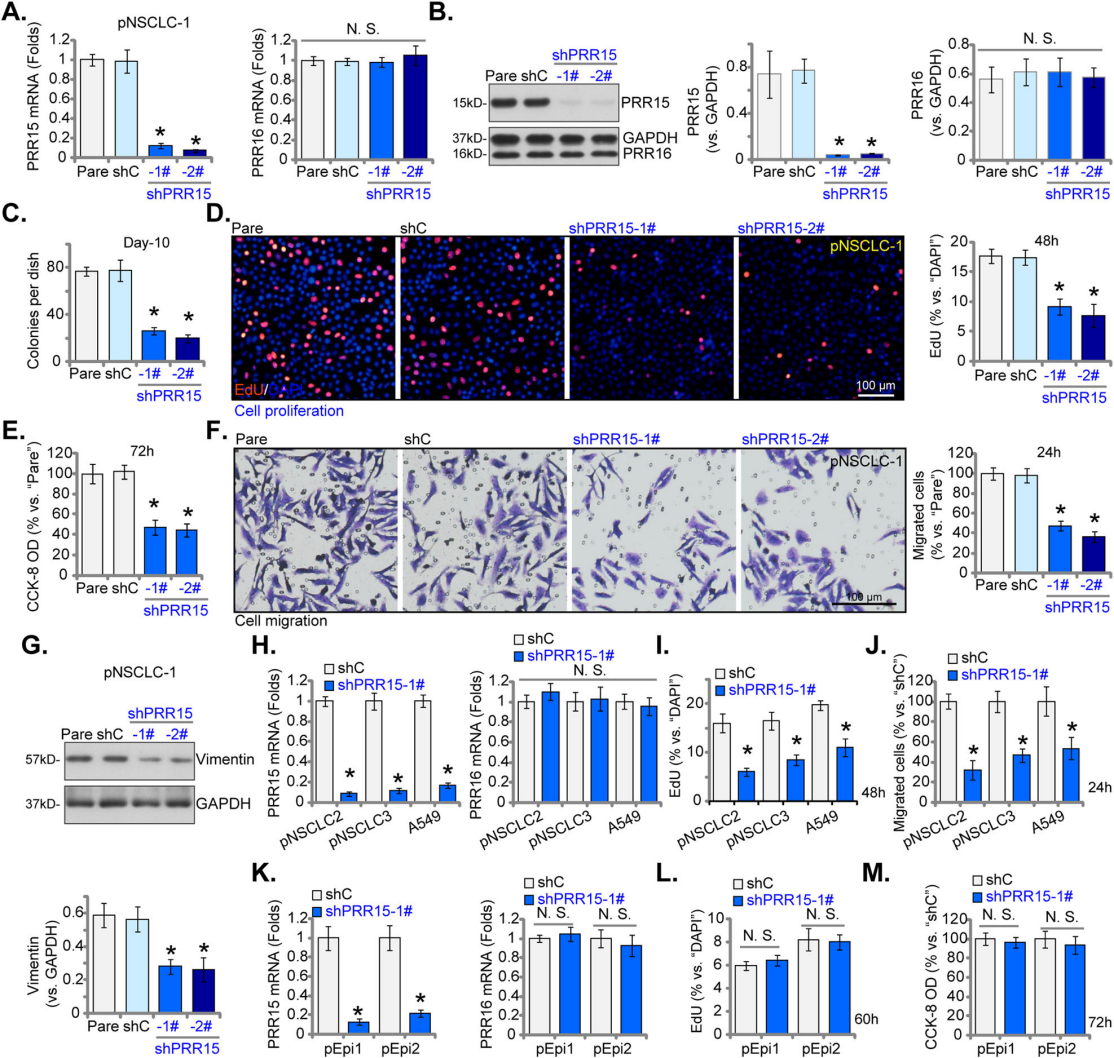
PRR15 shRNA triggers anti-cancer effects in NSCLC cells. Primary pNSCLC-1 cells were individually treated with the targeted PRR15 shRNAs (shPRR15-1# and shPRR15-2#, representing different sequences) or a control scramble shRNA (shC). The mRNA and protein levels of PRR15 and PRR16 were measured (**A**, **B**). Equal numbers of these cells were cultured for specific durations to evaluate various cellular functions, including colony formation (**C**), cell proliferation (percentage of EdU-incorporated nuclei, **D**), viability (CCK-8 OD, **E**) and cell migration (Transwell assays, **F**). Expression of Vimentin was also shown (**G**). Additionally, stable cells derived from other primary NSCLC cells (pNSCLC-2 and pNSCLC-3), the immortalized A549 cells, and lung epithelial cells (pEpi1 and pEpi2), expressing either shC or shPRR15-1# were established and examined for *PRR15* and *PRR16* mRNA expression (**H**, **K**). Equal numbers of these cells were cultured for specific durations to assess cell proliferation (percentage of EdU-incorporated nuclei, **I**, **L**), migration (**J**), and viability (CCK-8 OD, **M**) using the same methods. The data values are presented as the mean ± standard deviation (SD, *n* = 5). “Pare” refers to the parental control cells. Statistical significance was marked by * ***P*** < 0.05 compared to the “shC” cells, while “N.S.” denotes non-significant differences (***P*** > 0.05). These experiments were conducted five times, consistently producing similar results. Scale Bar = 100 μm.

To determine the role of PRR15 in NSCLC cells, shRNA-based methods were utilized to reduce its expression. Lentivirus containing PRR15 shRNA was introduced into pNSCLC-1 primary cells. Stable cells were established following puromycin selection. Two different shRNAs, shPRR15-1# and shPRR15-2# (targeting non-overlapping regions of PRR15), were employed. Both shRNAs effectively diminished *PRR15* mRNA (Fig. 4A) and protein (Fig. 4B) levels. Expression of PRR16, as a control, was however unchanged (Fig. 4A, B). The suppression of PRR15 via these specific shRNAs significantly curtailed the growth of pNSCLC-1 cells and inhibited their colony formation abilities (Fig. 4C). This knockdown also led to a decrease in the percentage of EdU-positive nuclei (Fig. 4D), supporting proliferation inhibition. Cell viability, measured by CCK-8 optical density (OD), was significantly diminished in PRR15-silenced pNSCLC-1 primary cells (Fig. 4E). Additionally, PRR15 shRNAs impeded the motility of pNSCLC-1 cells. The in vitro cell migration was markedly reduced, as shown by “Transwell” assays (Fig. 4F). In addition, expression of Vimentin, an epithelial-to-mesenchymal transition (EMT) protein important for NSCLC cell migration [38–40], was also decreased in PRR15-silenced pNSCLC-1 cells (Fig. 4G). The scramble non-sense control shRNA (“shC”) did not significantly alter PRR15/PRR16 expression (Fig. 4A, B) and the functional behaviors of pNSCLC-1 cells (Fig. 4C–G).

To evaluate the consistency of PRR15 knockdown in various NSCLC cells, lentivirus expressing shPRR15-1# was introduced into primary human NSCLC cells from different patients (pNSCLC-2 and pNSCLC-3) and into the immortalized A549 cell line. Puromycin selection was employed to establish stable cells. A substantial decrease in *PRR15* mRNA levels was observed in these NSCLC cells with shPRR15-1# treatment (Fig. 4H), while *PRR16* mRNA levels remained stable (Fig. 4H). Similar to the pNSCLC-1 cell results, PRR15 knockdown with shPRR15-1# consistently suppressed cell proliferation (indicated by reduced EdU-positive nuclei ratio, Fig. 4I) and inhibited the number of migrated cells (Fig. 4J) in both primary and immortalized NSCLC cells. In primary human lung epithelial cells, pEpi1 and pEpi2, PRR15 expression was suppressed using the same lentiviral construct shPRR15-1# (Fig. 4K). Despite the effective knockdown of *PRR15* (but not *PRR16*, Fig. 4K), there was no significant effect on cell proliferation (nuclear EdU incorporation) in these lung epithelial cells (Fig. 4L). Furthermore, cell viability tested via the CCK-8 assay showed no significant reduction due to shPRR15-1# treatment (Fig. 4M). Together, these results showed that PRR15 silencing by targeted shRNA induced robust anti-NSCLC cell activity.

### PRR16 shRNA triggers apoptosis activation in NSCLC cells

**Fig. 5:**
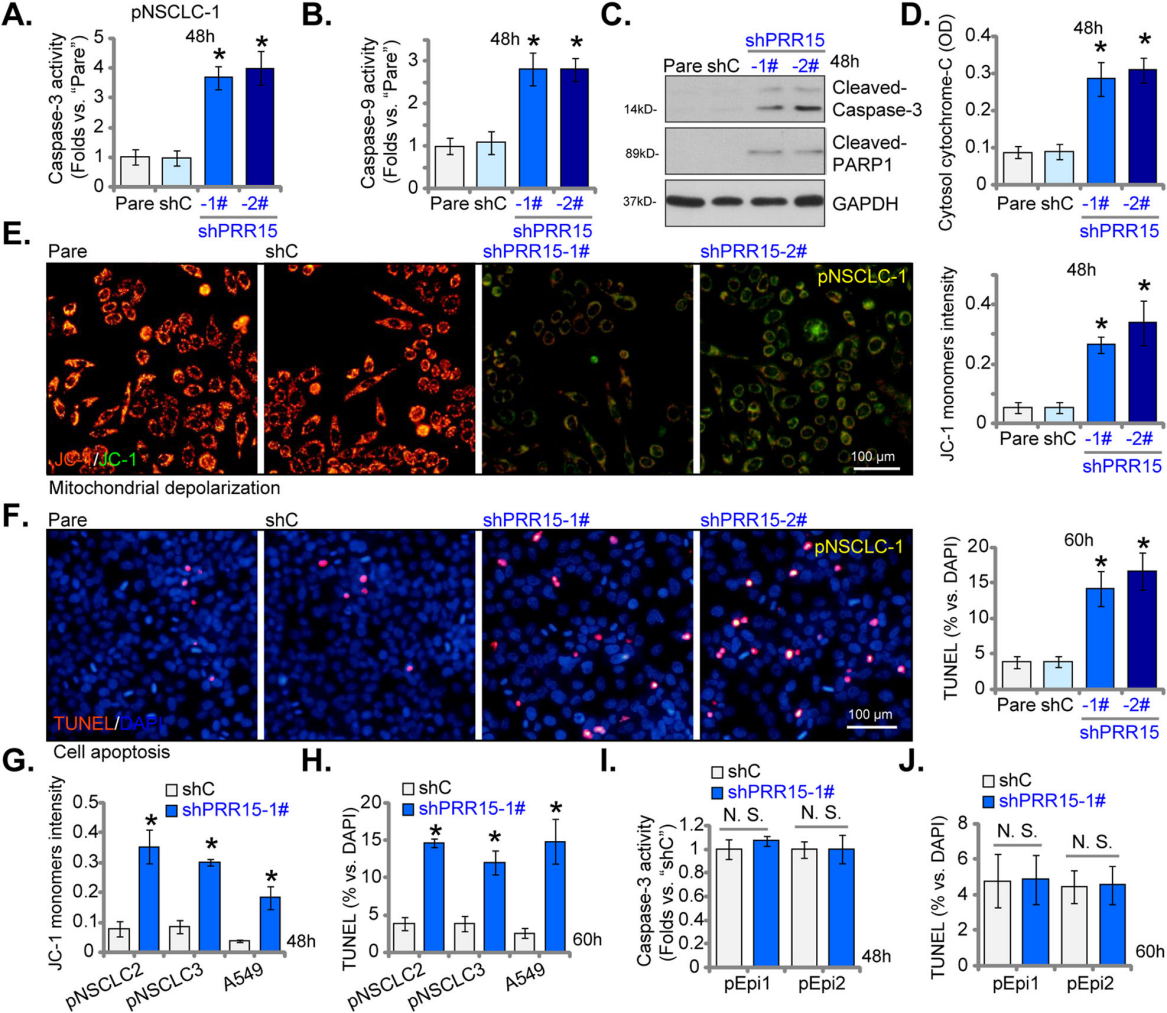
PRR15 shRNA triggers apoptosis activation in NSCLC cells. Primary pNSCLC-1 cells were individually treated with the targeted PRR15 shRNAs (shPRR15-1# and shPRR15-2#, representing different sequences) or a control scramble shRNA (shC). Equal numbers of these cells were cultured for specific durations to measure Caspase-3 and Caspase-9 activities (**A**, **B**), cleavages of apoptosis-related proteins (**C**), cytosolic cytochrome C levels using ELISA (**D**), and mitochondrial depolarization indicated by the accumulation of JC-1 green fluorescence monomers (**E**). Cell apoptosis was assessed by determining the percentage of TUNEL-positive nuclei (**F**). Additionally, stable cells derived from other primary NSCLC cells (pNSCLC-2 and pNSCLC-3), the immortalized A549 cells, and lung epithelial cells (pEpi1 and pEpi2), expressing either shC or shPRR15-1# were established and were cultured for specific durations to measure mitochondrial depolarization (**G**), Caspase-3 activity (**I**) and TUNEL-positive nuclei (**H**, **J**). The data values are presented as the mean ± standard deviation (SD, *n* = 5). “Pare” refers to the parental control cells. Statistical significance was marked by * ***P*** < 0.05 compared to the “shC” cells, while “N.S.” denotes non-significant differences (***P*** > 0.05). These experiments were conducted five times, consistently producing similar results. Scale Bar = 100 μm.

The above findings demonstrated that the shRNA-induced knockdown of PRR15 significantly suppressed cell viability, proliferation, and migration in both primary and immortalized NSCLC cells. To delve deeper into its functional role, we assessed its impact on cell apoptosis. pNSCLC-1 cells treated with shPRR15-1# or shPRR15-2# showed a marked increase in Caspase-3 and Caspase-9 activities (Fig. 5A, B). The shRNA-mediated silencing of PRR15 also triggered cleavages in Caspase-3 and poly(ADP-ribose) polymerase 1 (PARP1) within these cells (Fig. 5C). Moreover, cytosolic cytochrome-C levels, a crucial marker of apoptosis initiation, were elevated in pNSCLC-1 cells expressing PRR15 shRNA (Fig. 5D). Additionally, supporting mitochondrial depolarization, PRR15 silencing led to an accumulation of JC-1 green fluorescence monomers in pNSCLC-1 cells (Fig. 5E).

The silencing of PRR15 triggered heightened apoptosis in pNSCLC-1 cells, as shown by a significant increase in TUNEL-positive nuclei (Fig. 5F). Conversely, the shC control treatment did not induce Caspase and apoptosis activation in pNSCLC-1 cells (Fig. 5A–F). In other primary human NSCLC cells (pNSCLC-2 and pNSCLC-3), and in immortalized A549 cells, stable PRR15 knockdown via shPRR15-1#-expressing lentivirus (refer to Fig. 4) similarly resulted in mitochondrial depolarization (Fig. 5G) and apoptosis (Fig. 5I). The latter was evidenced by a rise in TUNEL-positive nuclei (Fig. 5H). However, in primary human lung epithelial cells (pEpi1 and pEpi2), PRR15 silencing did not enhance Caspase-3 activity (Fig. 5I) and increase TUNEL-positive nuclei (Fig. 5J). Together, these results showed that PRR15 silencing by targeted shRNA induced apoptosis in NSCLC cells.

### Significant anti-NSCLC cell effects of PRR15 knockout (KO)

**Fig. 6:**
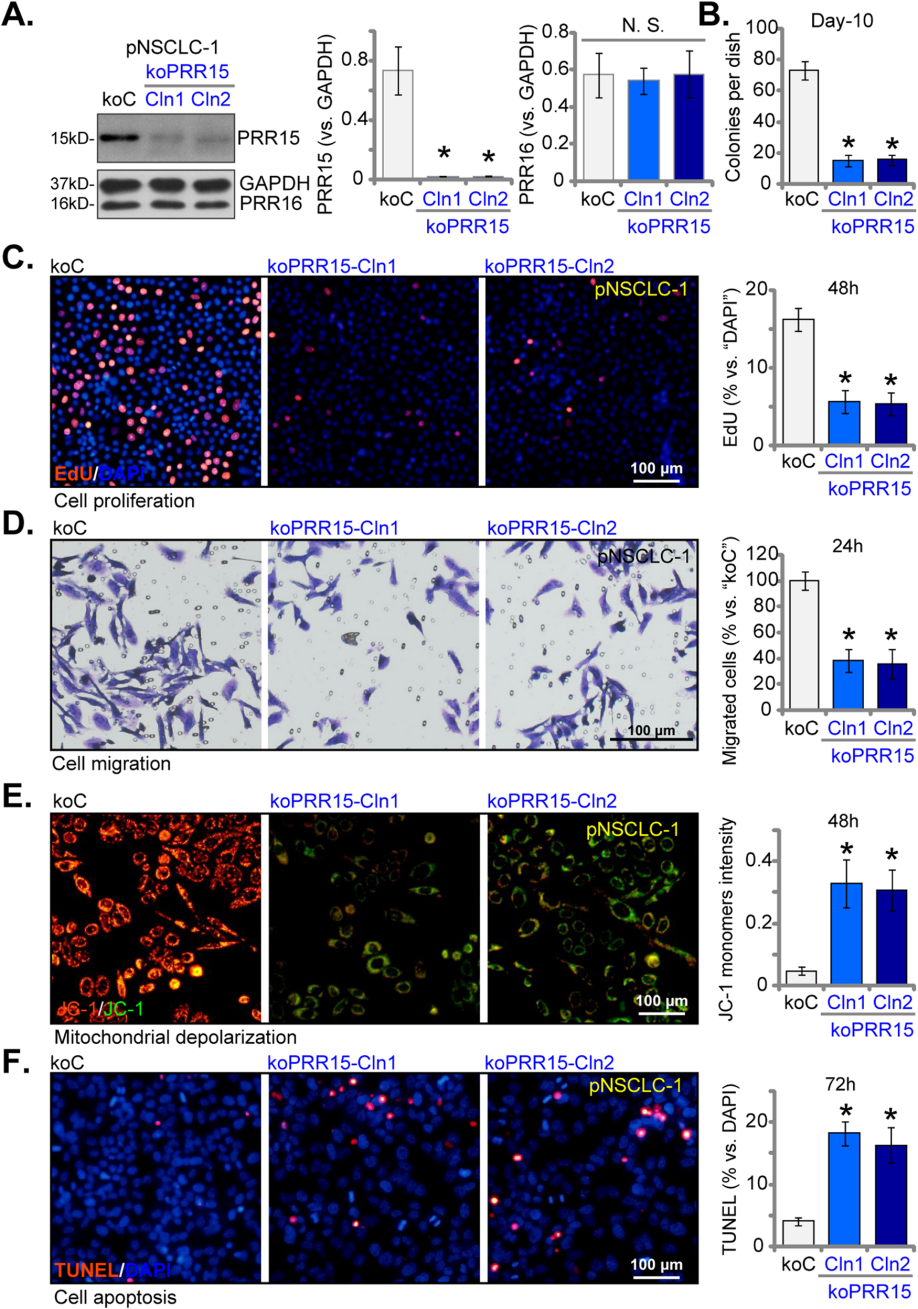
Significant anti-NSCLC Cell effects of PRR15 knockout (KO). The “koPRR15” pNSCLC-1 cells, consisting of sub-clones koPRR15-Cln1 and koPRR15-Cln2, were developed by transducing the CRISPR-PRR15-KO construct into Cas9-expressing pNSCLC-1 cells. Control cells, referred to as “koC”, were created by introducing the Cas9-expressing construct along with a CRISPR-KO control construct. Protein expression levels of PRR15 and PRR16 were shown (**A**). An equal number of these cells were cultured for specific periods to assess various cellular functions, including colony formation (**B**), cell proliferation (measured by the percentage of EdU-incorporated nuclei, **C**), in vitro cell migration (using “Transwell” assays, **D**), mitochondrial depolarization (assessed by JC-1 green fluorescence monomer intensity, **E**), and cell apoptosis (determined by nuclear TUNEL staining, **F**). The data values are presented as the mean ± standard deviation (SD, *n* = 5). Statistical significance was marked by * ***P*** < 0.05 compared to the “koC” cells, while “N.S.” denotes non-significant differences (***P*** > 0.05). These experiments were conducted five times, consistently producing similar results. Scale Bar = 100 μm.

To avoid potential off-target consequences from using shRNAs and achieve a total knockout (KO) of PRR15, we employed the CRISPR/Cas9 technique. We introduced lentivirus carrying the CRISPR/Cas9-PRR15-KO puro-construct into Cas9-expressing pNSCLC-1 cells. Following puromycin selection, stable cells were formed and were then screened for PRR15 KO. This led to the creation of single-cell-derived NSCLC-1 PRR15 KO colonies: “koPRR15-Cln1” and “koPRR15-Cln2”. Western blotting analysis confirmed the absence of PRR15 protein in koPRR15 pNSCLC-1 cells (Fig. 6A), with PRR16 expression remained unchanged (Fig. 6A). Functional tests demonstrated that PRR15 KO significantly suppressed the proliferation of pNSCLC-1 cells, indicated by a profound reduction in colony formation (Fig. 6B) and fewer EdU-positive nuclei (Fig. 6C). Furthermore, the in vitro migration (Fig. 6D) capability of the koPRR15-Cln1 and koPRR15-Cln2 pNSCLC-1 cells was markedly reduced. The KO of PRR15 also caused mitochondrial depolarization, leading to increase in JC-1 green fluorescence monomers in the pNSCLC-1 cells (Fig. 6E). Additionally, apoptosis was observed in PRR15 KO pNSCLC-1 cells, as evidenced by a higher nuclear TUNEL ratio (Fig. 6F). These results further supported a potential function role of PRR15 in NSCLC cell progression.

### PRR15 overexpression augments NSCLC cell growth

**Fig. 7:**
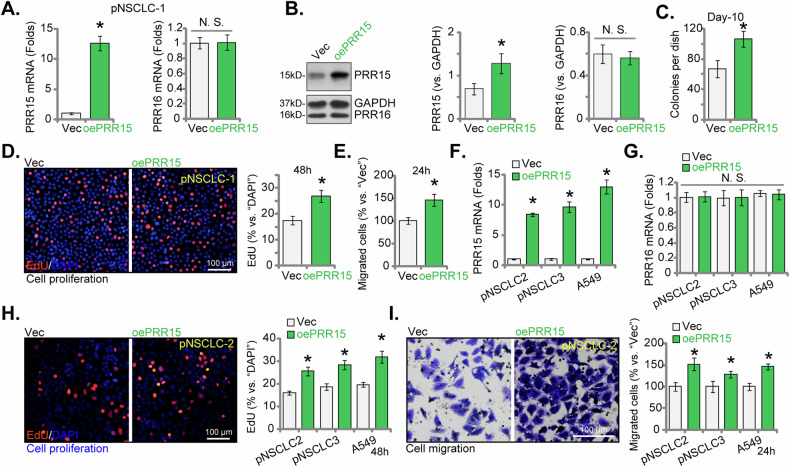
PRR15 overexpression augments NSCLC cell growth. The table cells derived from primary NSCLC cells (pNSCLC-1, pNSCLC-2 and pNSCLC-3) or the A549 immortalized cells, expressing the lentiviral PRR15 construct (“oePRR15”), along with those containing the empty vector (“Vec”), were generated for analysis of PRR15 and PRR16 expression (**A**, **B**, **F**, **G**). Equal numbers of these cells were cultured for specific durations to evaluate various cellular functions, including colony formation (**C**), cell proliferation (measuring the percentage of EdU-incorporated nuclei, **D**, **H**) and in vitro cell migration (“Transwell” assays, **E**, **I**). The data values are presented as the mean ± standard deviation (SD, *n* = 5). Statistical significance was marked by * ***P*** < 0.05 compared to the “Vec” cells, while “N.S.” denotes non-significant differences (***P*** > 0.05). These experiments were conducted five times, consistently producing similar results. Scale Bar = 100 μm.

The findings detailed above convincingly illustrated the significant anti-cancer effects resulting from the silencing or KO of PRR15 in both primary and immortalized NSCLC cells. This prompted us to hypothesize that overexpression of PRR15 might produce the opposite effects, potentially promoting NSCLC cell growth. Next, we introduced the lentivirus containing the PRR15-expressing construct into pNSCLC-1 cells. Following puromycin selection, we established stable cells (“oePRR15”). Compared to control pNSCLC-1 cells carrying an empty vector (“Vec”), oePRR15 cells exhibited a substantial upregulation in *PRR15* mRNA (Fig. 7A) and protein (Fig. 7B) levels. PRR16 expression remained unchanged (Fig. 7A, B). PRR15 overexpression significantly enhanced the proliferation of pNSCLC-1 cells, as evidenced by increased colony formation (Fig. 7C) and a higher percentage of EdU-positive nuclei (Fig. 7D). Additionally, PRR15 overexpression accelerated in vitro cell migration (Fig. 7E) in pNSCLC-1 cells.

Subsequently, we introduced the lentivirus containing the PRR15-expressing construct into additional primary NSCLC cells (pNSCLC-2 and pNSCLC-3) as well as the immortalized A549 cells. After puromycin selection, stable cells, “oePRR15”, were established. In these NSCLC cells, there was a significant elevation in *PRR15* mRNA levels (Fig. 7F), while *PRR16* mRNA levels remained unchanged (Fig. 7G). Overexpression of PRR15 increased cell proliferation, as demonstrated by the enhanced incorporation of EdU in the nuclei of NSCLC cells (Fig. 7H). Moreover, PRR15 overexpression led to accelerated in vitro cell migration in both primary and immortalized NSCLC cells (Fig. 7I). These data provide compelling evidence for the critical role of PRR15 in the progression of NSCLC.

### PRR15 is important for Akt-mTOR activation in NSCLC cells

**Fig. 8:**
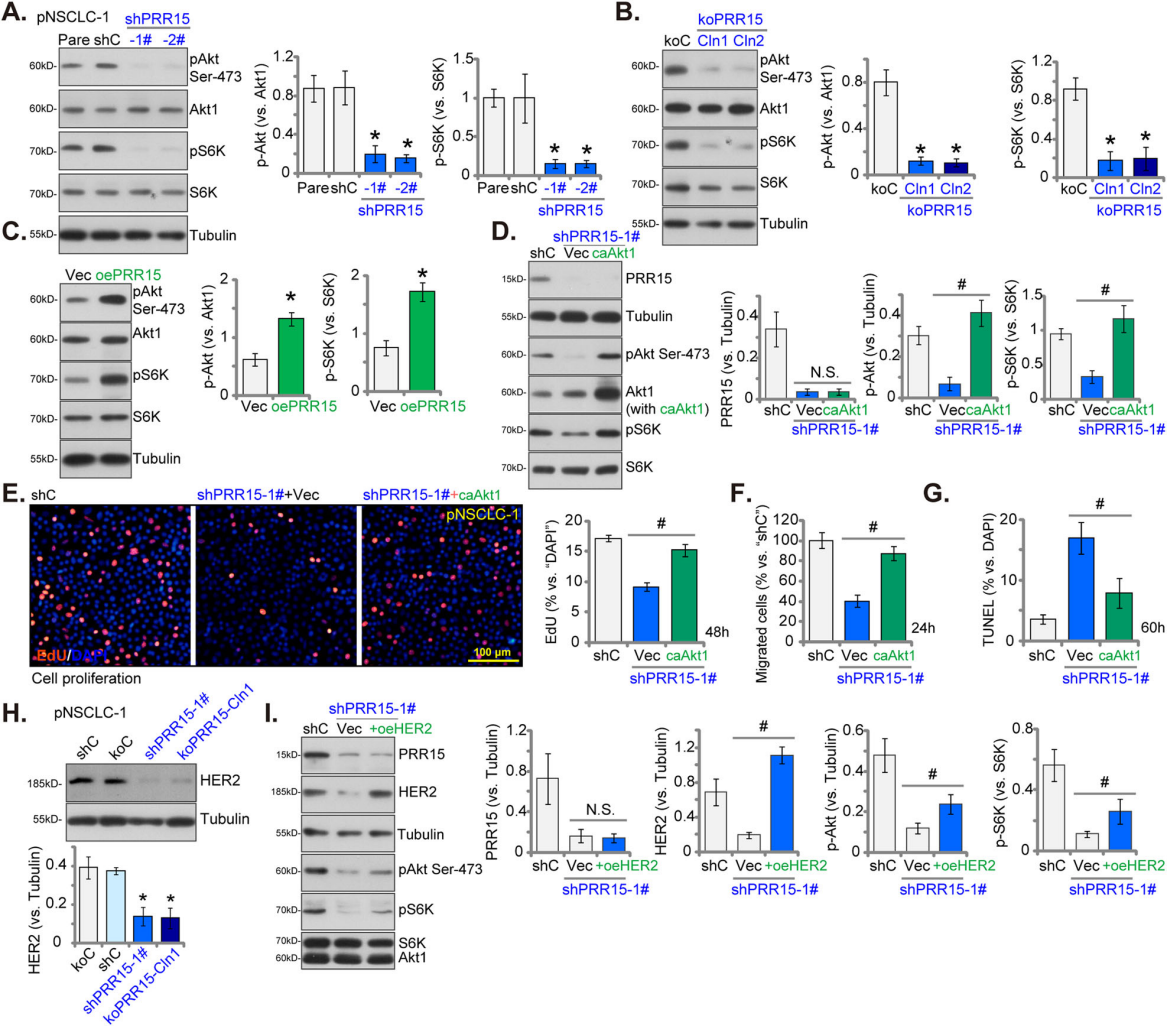
PRR15 is important for Akt-mTOR activation in NSCLC cells. The primary pNSCLC-1 cells with the PRR15 shRNA (shPRR15-1# or shPRR15-2#), a control scramble shRNA (shC), the koPRR15 pNSCLC-1 cells, consisting of sub-clones (koPRR15-Cln1 or koPRR15-Cln2), the “koC” control cells, pNSCLC-1 cells expressing the lentiviral PRR15 construct (“oePRR15”) or empty vector (“Vec”) were cultured and expression of listed proteins was tested (**A**–**C** and **H**). The shPRR15-1#-expressing pNSCLC-1 cells were stably transduced with either a constitutively-active S473D mutant Akt1 (caAkt1) or an empty vector (“Vec”), and listed proteins were shown (**D**). These cells were then cultured for specified time periods, and cell proliferation, migration, and apoptosis were measured using EdU-nuclei staining (**E**), “Transwell” assays (**F**), and TUNEL-nuclei assays (**G**), respectively, with results quantified. The shPRR15-1#-expressing pNSCLC-1 cells were stably transduced with either a HER2-overexpression construct (“oeHER2”) or an empty vector (“Vec”), and listed proteins were shown (**I**). The data values are presented as the mean ± standard deviation (SD, *n* = 5). Statistical significance was marked by * ***P*** < 0.05 compared to “shC”/“koC”/“Vec” cells. ^**#**^ ***P*** < 0.05. “N.S.” denotes non-significant differences (***P*** > 0.05). These experiments were conducted five times, consistently producing similar results. Scale Bar = 100 μm.

Given the established role of Akt-mTOR overactivation in the progression of NSCLC, our research sought to investigate the potential influence of PRR15 on Akt-mTOR activation within NSCLC cells. In pNSCLC-1 cells, the silencing of PRR15, achieved through shPRR15-1# and shPRR15-2# (shown in Fig. 4), led to a marked decrease in the phosphorylation of Akt (at Ser-473) and S6K (at Thr-389) (Fig. 8A). Importantly, the overall protein levels of Akt1 and S6K remained constant in these cells (Fig. 8A). Additionally, in PRR15 KO NSCLC-1 cells, including koPRR15-Cln1 and koPRR15-Cln2 (shown in Fig. 6), Akt and S6K phosphorylation was also significantly reduced (Fig. 8B). Once again, total Akt1 and S6K levels were unaffected (Fig. 8B). In contrast, pNSCLC-1 cells with overexpressed PRR15 (oePRR15, shown in Fig. 7) exhibited increased Akt-S6K phosphorylation levels (Fig. 8C). These results underscore the crucial role of PRR15 in promoting Akt-mTOR activation within NSCLC cells.

To elucidate the link between PRR15-induced NSCLC cell progression and the activation of the Akt-mTOR pathway, we introduced a constitutively-active mutant of Akt1 (S473D, “caAkt1”) into PRR15-silenced pNSCLC-1 cells (shPRR15-1#). As shown, caAkt1 successfully restored Akt-S6K phosphorylation without affecting PRR15 protein expression in shPRR15-1#-expressing pNSCLC-1 cells (Fig. 8D). Consequently, in pNSCLC-1 cells, caAkt1 significantly mitigated the inhibition of proliferation caused by PRR15 silencing (tested via nuclear EdU assays, Fig. 8E), reduced the decline in migrated cell number (Fig. 8F) and decreased apoptosis (Fig. 8G) triggered by PRR15 downregulation. These findings strongly suggest that promoting Akt-mTOR pathway activation is a key mechanism of PRR15-driven progression of NSCLC cells.

An earlier study demonstrated that PRR15 inhibited Akt-mTOR activation in triple-negative breast cancer (TNBC) cells [41], suggesting a context-dependent role for this protein. In NSCLC cells, it is plausible that PRR15 regulates different mechanisms to promote Akt-mTOR activation. HER2, absent in TNBC, is important for the activation of the PI3K/Akt/mTOR pathway in NSCLC cells [42, 43]. Our findings reveal that silencing PRR15 (shPRR15-1#) or knockout (koPRR15-Cln1) significantly reduced HER2 protein expression in pNSCLC-1 cells (Fig. 8H). Important, the restoration of HER2 protein levels via a lentiviral construct (“oeHER2”) partially rescued Akt-S6K phosphorylation in shPRR15-1#-expressing pNSCLC-1 cells (Fig. 8I). These results indicate that PRR15 may promote Akt-mTOR activation in NSCLC cells at least in part by modulating HER2 expression.

### PRR15 silencing inhibits the growth of NSCLC cell xenograft in nude mice

**Fig. 9:**
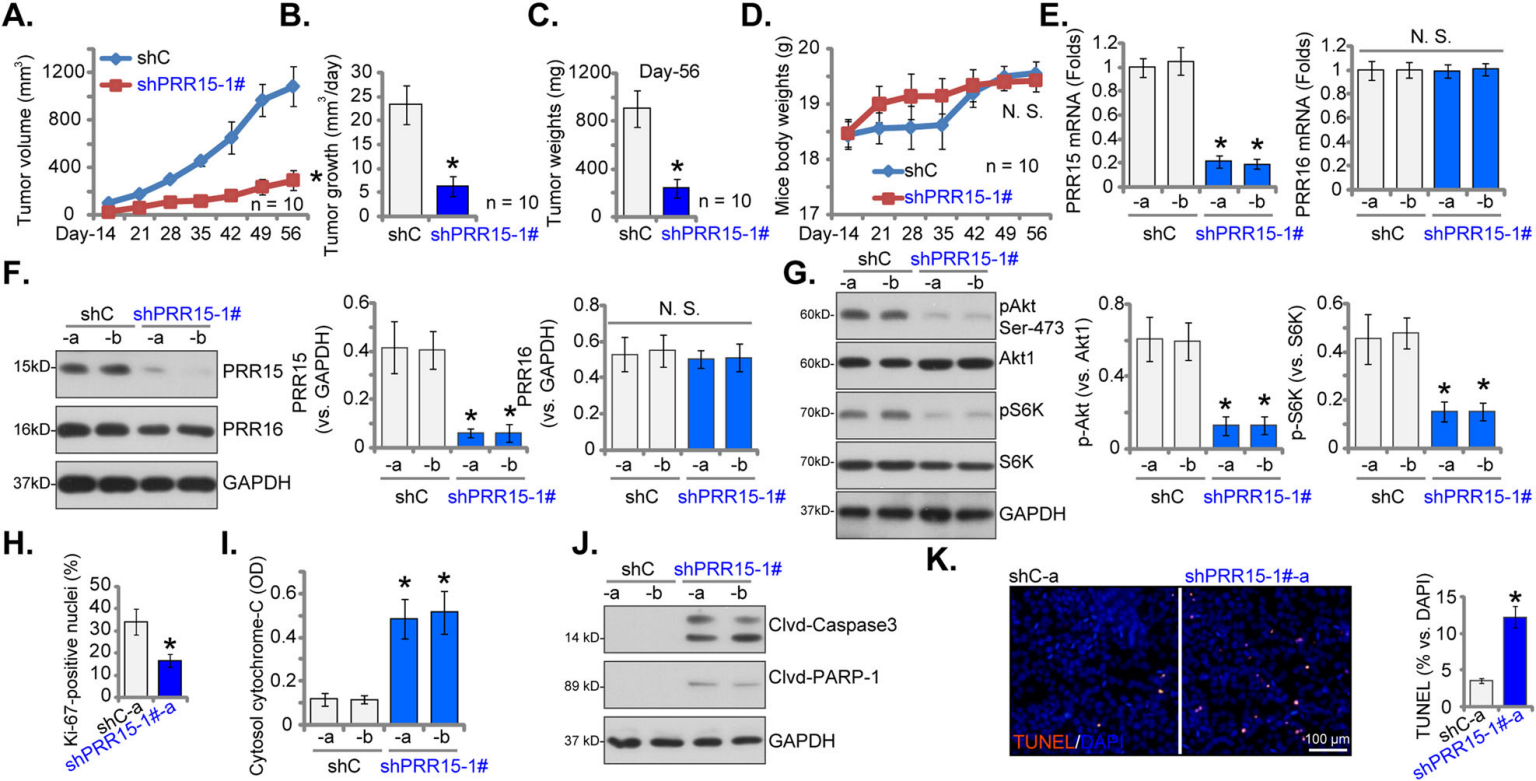
PRR15 silencing inhibits the growth of NSCLC cell xenograft in nude mice. Stable pNSCLC-1 cells, either expressing PRR15 shRNA (“shPRR15-1#“) or control shRNA (“shC”), were injected subcutaneously into the flanks of nude mice, with each mouse receiving eight million cells. Each group consisted of 10 mice (*n* = 10). Observations started 14 days after initial cell inoculation (“Day-12”). Tumor volumes (**A**, in mm³) and the body weights of the mice (**D**, in grams) were documented every seven days starting from Day-12 until Day-54. The daily growth rate of the tumors was analyzed (**B**). On Day-56, the tumors were excised surgically and their weights were measured (**C**). For further analysis, two tumors from each group (labeled as “shPRR15-1#-a” and “ shPRR15-1#-b”) were selected for protein and mRNA expression analysis in tissue lysates (**E**, **F**, **G**, **J**), and cytosol cytochrome C contents were assessed in the tissue lysates (**I**). Additionally, sections of the tumors underwent immunohistochemistry (IHC) staining for nuclear Ki-67, with Ki-67-positiven nuclei ratio quantified (**H**). TUNEL/DAPI fluorescence staining was also performed on the designated tissue sections (**K**). Data were presented as mean ± standard deviation (SD). Statistical significance was indicated by *P < 0.05 compared to the “shC” group. “N. S.” denotes non-statistically significant differences (***P*** > 0.05). For **A**–**D**, the sample size was 10 mice per group. In **E**–**K**, each tumor was sectioned into five pieces and analyzed individually.

To understand the role of PRR15 in NSCLC cell growth in vivo, we subcutaneously injected eight million pNSCLC-1 cells, transduced with either shPRR15-1# or shC, into nude mice flanks. Monitoring commenced 14 days post injection (“Day-14”). Tumor volume measurements every seven days, from “Day-14” to “Day-56”, showed significantly reduced growth in shPRR15-1#-expressing pNSCLC-1 xenografts compared to shC controls (Fig. 9A). Daily growth analysis, shown as mm^3^ per day, confirmed substantial tumor suppression in shPRR15-1# xenografts (Fig. 9B). On “Day-56”, all pNSCLC-1tumors were excised and weighed, revealing significantly lower masses in the shPRR15-1# group than in the shC group (Fig. 9C). Mouse body weights remained stable across both groups (Fig. 9D).

In shPRR15-1# pNSCLC-1 xenograft tissues, there was a significant decrease in *PRR15* mRNA (Fig. 9E) and protein (Fig. 9F) levels, confirming PRR15 silencing. While *PRR16* mRNA and protein expression levels were unchanged (Fig. 9E, F). PRR15 knockdown in pNSCLC-1 xenograft tumors resulted in a pronounced decrease in phosphorylated Akt and S6K, consistent with Akt-mTOR pathway suppression (Fig. 9G). This finding was corroborated by a significant reduction in Ki-67-positive nuclear ratio, signifying diminished intratumoral proliferation (Fig. 9H). In contrast, the cytosol cytochrome C levels were increased (Fig. 9I), accompanied by increased levels of cleaved Caspase-3 (Fig. 9J) and cleaved PARP-1 (Fig. 9J) in shPRR15-1#-expressing xenografts. Fluorescence staining assays in tumor slides revealed a higher percentage of TUNEL-positive nuclei in PRR15-silenced tumors (Fig. 9K), further supporting apoptosis induction. Thus, PRR15 silencing demonstrably compromised Akt-mTOR signaling, resulting in consequent suppression of proliferation, induction of apoptosis, and ultimately, impeded NSCLC xenograft tumor growth.

## Discussion

Targeted therapies for NSCLC have demonstrated clinical utility in specific patient subsets harboring actionable molecular alterations [44]. However, several limitations constrain their overall impact. The development of resistance mechanisms, including acquired mutations, activation of alternative signaling pathways, and intratumor heterogeneity, frequently undermines therapeutic efficacy [44]. Moreover, the accurate identification of predictive biomarkers remains elusive, hindering optimal patient selection and treatment stratification [44–46]. It is therefore an urgent need to identify novel therapeutic targets.

Our findings indicate that PRR15 is a potential novel therapeutic target and diagnostic biomarker for NSCLC. Elevated PRR15 expression was observed in NSCLC tissues compared to normal lung parenchyma, with a correlation between higher expression and adverse clinical outcomes. Single-cell RNA sequencing confirmed PRR15 overexpression within the malignant tumor cell population. Functional studies demonstrated that PRR15 depletion inhibited NSCLC cell proliferation and migration, while promoting apoptosis. Conversely, PRR15 overexpression exhibited pro-tumorigenic effects. In vivo studies further validated these findings, demonstrating that PRR15 knockdown suppressed tumor growth in xenograft models. These results strongly support the oncogenic role of PRR15 in NSCLC and its potential as a therapeutic target.

The Akt-mTOR pathway is a central signaling hub that orchestrates multiple oncogenic processes critical to NSCLC progression [14, 43, 47]. Its activation promotes unchecked cell proliferation, inhibits apoptosis, and stimulates angiogenesis, fostering tumor growth and metastasis [14, 43, 47]. Moreover, the pathway is implicated in metabolic reprogramming, enabling tumor cells to adapt to the nutrient-deprived tumor microenvironment. Akt-mTOR signaling confers resistance to various therapeutic modalities, including chemotherapy and targeted therapies, underscoring its pivotal role in treatment failure [43]. Consequently, targeting this pathway represents a promising therapeutic strategy for NSCLC, with the potential to improve clinical outcomes and enhance patient survival [43]. Multiple Akt-mTOR inhibitors, including GDC-0349, GDC-0941, BEZ-235, PQR620 and MTI-31, have demonstrated significant anti-tumor activity in NSCLC preclinical models [32, 48–51].

Hyper-activation of Akt-mTOR cascade in NSCLC is attributed to a complex interplay of genetic, epigenetic, and microenvironmental factors. Genetic alterations, such as activating mutations in *PIK3CA* and inactivating mutations in *PTEN*, directly contribute to pathway hyperactivation [8, 14, 43, 52]. Additionally, epigenetic modifications, including DNA methylation and histone alterations, can modulate the expression and activity of key pathway components [14, 43, 47]. Furthermore, aberrant receptor tyrosine kinase signaling, commonly observed in NSCLC, can indirectly activate Akt-mTOR through upstream signaling cascades [14, 43, 47]. The tumor microenvironment also plays a role, with factors like hypoxia, inflammation, and nutrient deprivation contributing to pathway dysregulation [14, 43, 47, 53].

While the precise mechanisms underlying Akt-mTOR hyperactivation in NSCLC remain elusive, recent investigations have implicated several key players in promoting this cascade. Mitochondrial proteins such as YME1 Like 1 (YME1L) and aarF domain containing kinase 2 (ADCK2), the sodium/myo-inositol co-transporter SLC5A3, and the SH2-domain containing protein SH2B1 have emerged as potential contributors to Akt-mTOR pathway dysregulation [30, 54–56]. Our findings establish a critical role for PRR15 in the activation of the Akt-mTOR pathway in NSCLC cells. We demonstrated that suppression of PRR15 expression through shRNA or CRISPR/Cas9-mediated KO led to a significant attenuation of Akt and S6K phosphorylation, key downstream effectors of Akt-mTOR signaling. Conversely, overexpression of PRR15 resulted in enhanced Akt-S6K signaling. Furthermore, rescue experiments revealed that restoration of Akt-mTOR activity through caAkt1 effectively abrogated the growth inhibitory effects induced by PRR15 knockdown. These findings were corroborated by in vivo studies demonstrating reduced Akt-mTOR activation in NSCLC xenograft tumors with PRR15 silencing. Collectively, our data strongly implicate PRR15 as a pivotal regulator of Akt-mTOR signaling in NSCLC tumorigenesis.

## Supplementary information

Figure S1.

## Data Availability

All data are available upon reasonable request.
