# Supplementary material for: The expression and functional role of proline-rich 15 in non-small cell lung cancer
Source: Cell Death Dis. 2025 Feb 10;16(1):83. doi: 10.1038/s41419-025-07373-x (PMC11811231; doi:10.1038/s41419-025-07373-x)

Figure S1. The uncropped blotting images of the study.

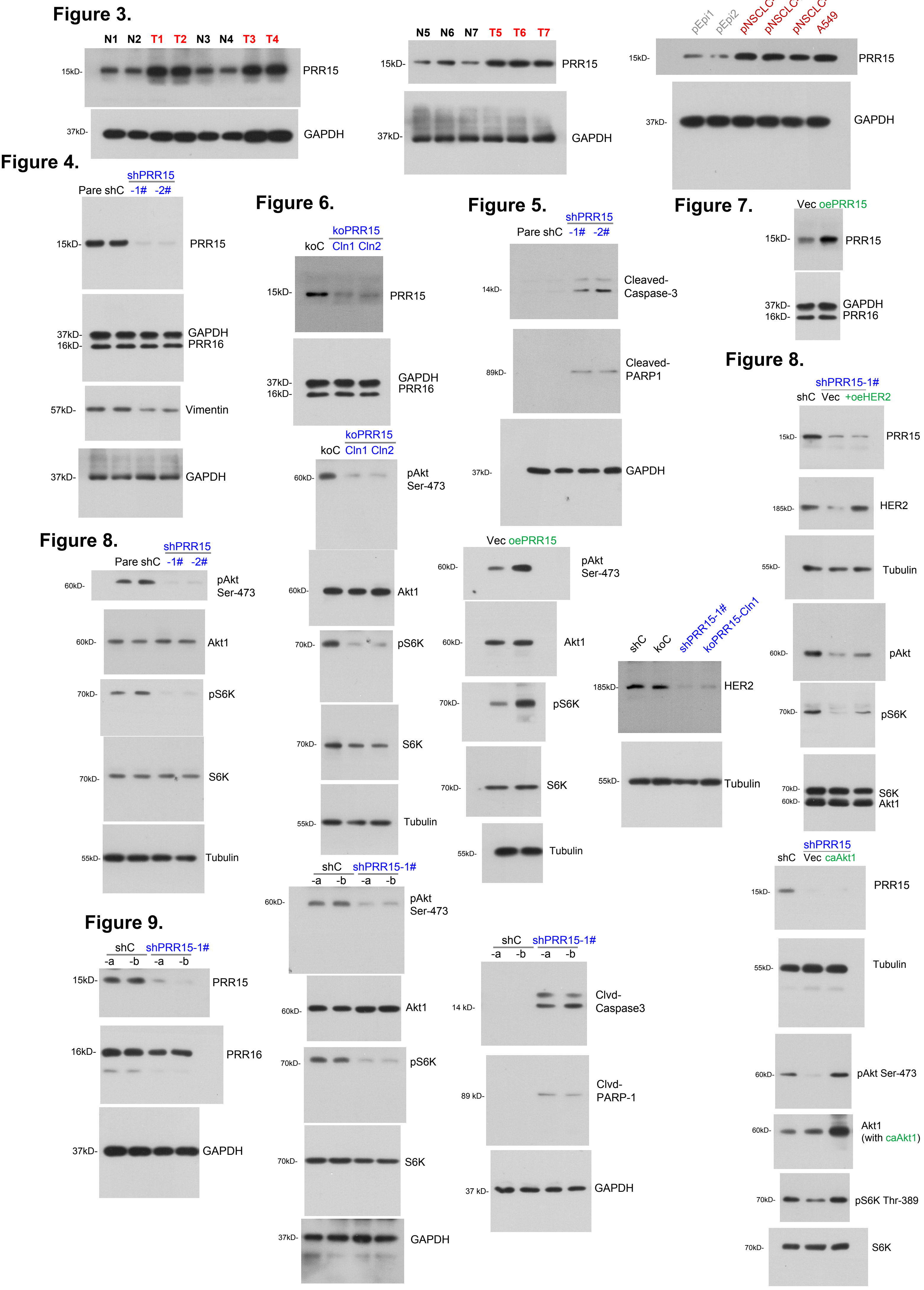

Supplement: Supplementary file 1 — Figure S1. [file 41419_2025_7373_MOESM1_ESM.pdf]
